# Morphological and Physiological Responses of Hybrid Aspen (*Populus tremuloides* Michx. × *Populus tremula* L.) Clones to Light *In Vitro*

**DOI:** 10.3390/plants11202692

**Published:** 2022-10-12

**Authors:** Toms Kondratovičs, Mārtiņš Zeps, Diāna Rupeika, Pauls Zeltiņš, Arnis Gailis, Roberts Matisons

**Affiliations:** Latvian State Forest Research Institute ‘Silava’, 111 Rigas Street., LV-2169 Salaspils, Latvia

**Keywords:** chlorophyll, leaf area, LED, micropropagation, shoot length

## Abstract

Micropropagation of fast-growing tree genotypes such as the hybrid aspen (*Populus tremuloides* Michx. × *Populus tremula* L.) is increasing. The efficiency of micropropagation depends on the luminaires, hence luminescent electric diodes (LED), which emit light of a narrow spectrum, are gaining popularity. Mostly, different LEDs are combined to increase the photosynthetic efficiency. However, light also acts as an environmental signal, which triggers specific responses in plants, which are genotype specific, and regarding hybrid aspen, are likely affected by heterosis. In this study, morphological and physiological responses of clones of hybrid aspen with contrasting field performance to the spectral composition of illumination were studied in vitro. Among the 15 variables measured, area of leaves and concentration and ratio of chlorophyll a and b explained most of the variance (58.6%), thereby linking a specific combination of traits to productivity. These traits and their responses to light were affected by heterosis, as indicated by the clone-treatment interaction, particularly for the clone’s moderate productivity. The top-performing clones were little sensitive to illumination due to efficient photosystems. Nevertheless, illumination with wider spectral composition had generally positive effects on plantlet performance. Accordingly, clone-specific illumination protocols and luminaries capable of it are advantageous for the efficiency of micropropagation of hybrid aspen.

## 1. Introduction

Under the growing demand for timber, renewable energy resources, and carbon sequestration, the relevance of fast-growing stress-tolerant tree genotypes, as a mean for improving productivity and sustainability of forests, is increasing [1,2]. Under temperate and hemiboreal conditions, hybrid aspen (*Populus tremula* L. × *P. tremuloides* Michx.), which shows superior growth compared to local and introduced tree species [3] is one of the most promising alternatives for multipurpose commercial forestry, providing services from timber production to bioremediation [4,5,6]. The superiority of the hybrids can be stable across different growing condition because of heterosis [7,8], which interactively amplifies the expression of certain traits exceeding those of parental plants [9]. Such stability of phenotype has been linked to heterozygosity due to the hybridization of naturally evolved inbreeds [10]. In general, four main types of heterosis have been identified (dominance, overdominance, pseudo-overdominance, de-novo interlocus inter-action or epistasis), which however, interfere, hence, the genetics of the resulting traits are complex [11]. Accordingly, the effect of heterosis is difficult to predict just basing on the phenotypes of parental plants [9,10,11,12].

For hybrids of trees, the effects of heterosis have mostly been studied for growth traits due to their economic relevance [8]. For hybrid aspen, the effect of heterosis regarding growth traits has been linked to the loss of sensitivity (increased plasticity) to local conditions as plants invest resources in growth [8,12], likely by the costs of production of defense substances and cold hardiness, which then have long-term effects on competitiveness and survival [8,13,14]. From the evolutionary perspective, such properties might not be viable as they mostly shatter the trade-offs between growth and defense without long-term advantage, however, from the commercial perspective under managed environment they can be highly beneficial [7,15]. Pest/disease resistance and cold hardiness can be improved by breeding techniques, presuming independent genetic controls [15,16]. Nevertheless, the effects of heterosis likely influence a spectrum of traits, for instance due to rapid growth of hybrids, photosynthetic apparatus, root system, etc. must be concomitantly enhanced (e.g., larger leaves, higher concentration of chlorophyll, etc.) to provide sufficient assimilation of carbohydrates, thus, maintaining productivity [14,17]. The effects of heterosis are a result of non-additive inheritance, which is expressed in the F1 generation [14,17], hence vegetative propagation is needed to maintain it [15].

Clonal micropropagation is the main method for commercial, as well as scientific proliferation of genotypes of hybrid aspen [18], which is a resource and knowledge intensive process. Illumination plays a key role in commercial clonal micropropagation, particularly in terms of energy efficiency and hence the costs [19]. In the last decade, luminescent electric diode (LED) luminaries have been replacing commonly used fluorescent tubes due to reduced thermal radiation, superior energy efficiency, longevity, compact size, and versatility in terms of combination [20,21]. Although, LED emit light of narrow, as well as of broad spectra, there is still necessity for tailored luminaries capable of specific illumination thus aiding efficiency of vegetative propagation [21,22,23].

For vascular plants, light is simultaneously an energy source and environmental signal allowing assessment of local conditions and inducing responses to maximize long-term performance/survival [24]. Such signaling effects of light composition can be explicit under controlled environments, particularly in vitro [24]. The light signals are captured by several photoreceptors [25,26,27,28,29,30]. Phytochromes are sensitive to red (R) and far-red (FR) part of the spectrum affecting growth, plant movement, development of stomata, and initiate antioxidative systems [27,28], as a response to open conditions [29]. However, monochromatic R light has been shown to reduce photosynthetic capacity [30]. Blue (B) and ultraviolet (UV-A) light is absorbed by cryptochromes, which regulate chlorophyll concentration and leaf area [30,31,32]. Leaf and organelle movements are affected by phototropines, which react to Blight [26].

Plants have evolutionally adapted to the full light spectrum, deviations from which initiate specific responses [33]. Accordingly, the tradeoffs of signals from different photoreceptors are the triggers of physiological and structural responses [26,33,34], implying interacting effects of light of different wavelengths [35], and bias of the responses to monochromatic lights [36,37]. Such interactions allow efficient shade avoidance under a variety of local conditions [38,39,40], adjusting physiology and morphology according to canopy status [41]. The R and B ratio (R:B) has been identified as the main light signal for dicots, resulting in physiological, functional and morphological adjustments [30,37,42]. Furthermore, green (G) light, which penetrates canopy most efficiently providing energy for lower leaves, as well as understory plants, stimulates CO_2_ uptake and modulates the responses to R:B signals, stimulating shade tolerance [38,43].

The general responses of vascular plants to light of different wavelengths and their interactions have been identified [34,44]; however, there is variability among taxa and even populations due to local adaptation and legacy effects of environment [45,46,47]. The responses to light composition have been studied in model genera such as *Arabidopsis*, or widely cultivated cultures such as *Zea*, *Solanum*, *Oryza*, etc., which are herbaceous annual plants [46,47,48,49]. Trees, which are long-living organisms have received some attention [19,50], still with advances of clonal micropropagation of forest regenerative material, these issues are becoming topical [19]. Regarding the F1 hybrids, the responses to light signals are likely influenced by the complex effects of clone and heterosis [51], which imply the necessity for clone-specific fine-tuning of illumination conditions for maximum efficiency of micropropagation process.

From the practical point of view, the alterations of plantlet morphology by illumination can affect manipulations of plantlets in vitro [21], and their further development during the ex vitro and subsequent stages. In this regard, thicker stems and larger photosynthetic apparatus are preferable [52]. Illumination can also have a legacy effect on morphology and physiology of plantlets affecting the efficiency of propagation during the latter stages [34,35,36,37]. Such legacy effects have been demonstrated for plantlet leaves, which adjust to light conditions during formation of growth initials [53]; however, considering longevity, hence, the more conservative growth strategy of trees, such effects are likely for variety of traits. Regarding hybrid aspen, plantlet and, particularly, leaf morphology and physiology has been explicitly affected by heterosis [8], hence responses to illumination are expected to differ among hybrids reflecting to G×E effects [15].

The aim of this study was to evaluate responses of morphology and physiology of plantlets of aspen hybrid clones differing by productivity to artificial illumination of different composition. We hypothesized that clones would show specific responses, however, the responses would depend based on productivity, implying that similar sets of traits boost growth.

## 2. Results

The 15 morphological and physiological variables measured for plantlets of hybrid aspen in vitro showed uneven effects of the studied light treatments and clones, as indicated by the differing coefficients of variation (Table 1). The degree of variation, however, ranged similarly for the morphological and physiological variables. The lowest variation (coefficient of variation <0.20) was estimated for maximum quantum yield efficiency F_m_/F_v_, the ratio of chlorophyll a and b (CHL_A/B_), as well as for the total shoot length (TSL). In contrast, the highest variability (coefficient of variation >0.40) was estimated for total leaf area and, particularly, performance index of photosystem (PI), and activity of peroxidase (POX). The distribution for all variables was symmetric, as the mean values and medians were comparable.

**Figure 1:**
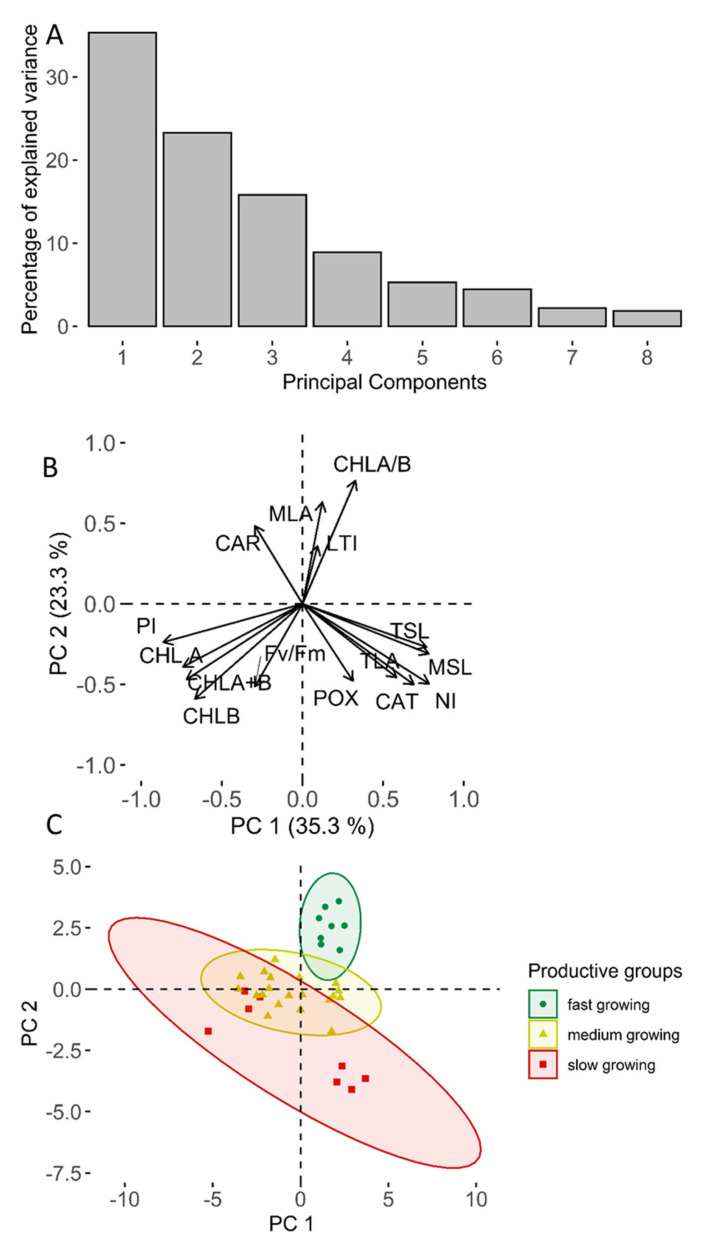
Relative variance explained by the principal components of the studied anatomical and physiological variables (means of experimental units, i.e., clone by treatment) of hybrid aspen (**A**), loadings of the variables (**B**) and scores of the experimental units (**C**). In C, ellipses indicate 95% confidence intervals of scores for clones with contrasting filed performance (high, intermediate, and low). Variable codes: NI—number of internodes; LTI—length of third internode; MSL—main shoot length; TSL—total shoot length; MLA—mean leaf area; TLA—total leaf area; CAR—concentration of carotenoids; CHLA—concentration of chlorophyll a; CHL B—concentration of chlorophyll b; CHLA+B—total chlorophyll concentration; CHLA/B—chlorophyll a and b ratio; Fv/Fm—maximum quantum yield efficiency; PI—performance index; CAT—catalase activity; POX—peroxidase activity.

The studied dataset of morphological and physiological variables contained three significant principal components with eigenvalues exceeding 1.0, while the first two components explained ca. 59% of the variances of the variables (Figure 1A). Most of the studied variables correlated with both principal components, with none showing explicit correlation with a single component. The loading of the first two principal components indicated that three correlated groups of variables could be distinguished, implying possibility for data reduction without substantial loss of crucial information. One of these groups consisted only of variables describing physiological performance of plantlet photosystem, while the other two groups had both physiological and morphological variables. The correlation among the groups, however, was intermediate implying complex source of variation of morphology and physiology of the plantlets. Considering the correlation between the variables and principal components, the total concentration of chlorophyll (a and b), the ratio of chlorophyll a and b, and the total area of leaves (per plantlet) were selected for the representation of the effects of clone and spectral composition of light (studied light treatments) on plantlets of hybrid aspen.

The scores of the first two principal components distinguished response of the clones of hybrid aspen to spectral composition of light according to their field performance indicting complex effects of heterosis on the studied traits of plantlets (Figure 1C). The ordination of experimental units (clones by light spectrum) according to the scores of the first two components showed that the productive clones had specific set of physiological and morphological traits, as indicated by substantially narrower range of points. In contrast, the set of the main traits of the low-productive clones was highly variable, yet differed from the productive ones, as indicated by a wider and non-overlapping envelope. Supporting the assumption, the combination of traits of the clones with intermediate performance, as indicated by the envelope of points, was intermediate and overlapped with the others, particularly with the low-productive ones. Accordingly, the ordination suggested that specific settings of heterosis of different traits was responsible for field performance of hybrid aspen. The ordination of the experimental units according to their first two principal components, however, did not distinguish points according to light composition, likely indicating effect of the studied treatment to be secondary with the respect to clone.

The plantlets of hybrid aspen in vitro were sensitive to the spectral composition of illumination, as indicated by the strictly significant effect of the studied light treatments on the morphological and physiological variables representing the principal components of the dataset (Table 2). For these variables, particularly CHL_A/B_, the individual effect of the clone exceeded that of the light treatment several times. The responses of these variables were clone specific, as indicated by the significant fixed effect of treatment by clone interactions, particularly for total leaf area (TLA; Table 2), indicating genetic differences in sensitivity to illumination. For the selected variables, the performance of the statistical models was high (marginal R^2^ ≥ 0.63); although biological responses have been analyzed. Negligible effects of experimental design in terms of inter class correlation of experimental design (inter class correlations (ICC) ~ 5%) were estimated by the variances of random effects for the morphological variable (TLA). However, for the selected physiological variables total concentration chlorophyll a and b (CHL_A+B_) and CHL_A/B_, which were estimated based on the plant material propagated collectively in jars, the random effect was substantial (ICC ≥ 0.56%), suggesting influence of interactions among the plantlets. Nevertheless, these dependencies were accounted for when estimating the fixed effects by the statistical model.

**Figure 2:**
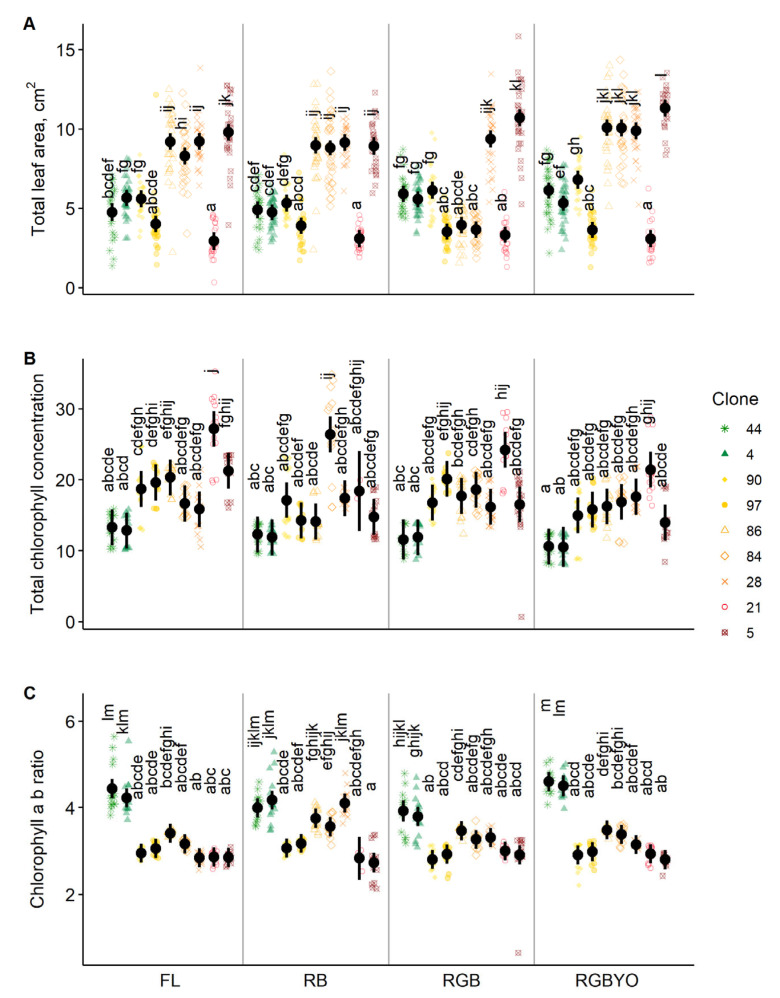
The estimated marginal mean values and their 95% confidence intervals (black dots and whiskers) for total area of leaves per plantlet (**A**), chlorophyll a and b ratio (**B**), and the total concentration of chlorophyll a and b (**C**) according to light treatments and clone of hybrid aspen in vitro. Similar letters above the data indicate lack of significant differences at α = 0.05. Points in the background depict the mean values of propagation jars/test tubes. The clones are ranged according to their field performance from the highly productive (clones No.: 4 and 44) to low productive (clones No.: 21 and 5) clones.

The individual effect of light treatment on TLA was expressed as a slight increase under the RGBYO, and as a slight decrease under RGB (Figure 2A), which differed by the quantity of yellow (Y) and orange (O) light (Table 3). The CLH_A+B_ was decreased under RGBYO and RB, which had lower proportion of G and higher proportion of R light, compared to RGB and, particularly, FL (Figure 2B), thus suggesting sensitivity to B:G ratio (Table 3). In contrast, higher values of HL_A/B_ were estimated under increased RGBYO and, particularly, RB treatments, supporting the sensitivity of plantlets to the B:G ratio (Figure 2C; Table 3).

The expressions of clone by treatment interactions were specific for the variables, yet generally indicated relationships with field performance of the clones. The responses of the productivity groups were not always homogeneous, indicating complex regulation of the studied physiological and morphological traits of plantlets in vitro (Figure 2). Nevertheless, the effect of the estimated clone by light treatment interaction indicated that the top-performing clones showed little to no sensitivity to light treatment according to TLA and CHL_A+B_. Regarding TLA, which represents the quantity of photosynthetic apparatus, the clone by treatment interaction was explicit for the clones with intermediate and low field performance (Figure 2A). Significantly, higher TLA was estimated for the intermediate clones No.: 28, 84, and 86, and low performing clone No. 5 under FL, RB, and RGBYO treatments. The intermediate clones No. 84 and 86, showed decreased TLA under RGB treatment, suggesting sensitivity to the amount of R, Y, and O light, as well as that of B and G light ratio (Table 3). The intermediate clone No. 97 and low productive clone No. 21 showed explicit reduction TLA under all but the RGB treatment. In contrast, the low productive clone No. 5 showed significantly higher TLA under RGB and, particularly, RGBYO treatments. Both the top-performing clones (No. 4 and 44), were estimated to have intermediate and mutually similar TLA irrespectively of light treatment (Figure 2A).

A general pattern of negative relationships between the CHL_A+B_, which represents the extent of photosystem and resources allocated to it, and field performance of clone was estimated under all but the RB treatments, under which the intermediate Clone No. 84 had significantly higher concentration of chlorophyll (Figure 2B). Irrespective of treatment, the top-performing clones were similarly estimated with the lowest CHL_A+B_. The light treatment specific responses, in contrast, were estimated for the intermediate and low productive clones. The low productive clone No. 21 showed increased CHL_A+B_ under FL, RGB, and RGBYO treatments, which contained G and R light. The low productive clone No. 5 showed intermediate values under all, but the RGBYO, under which a decreased CHL_A+B_, comparable to that of the top-performing clones, was estimated (Figure 2B).

The CHL_A/B_, which represents an acclimation of photosystem to illumination, tended to show positive relationships with the field performance of clones, as the highest and lowest values were estimated for the top-performing and low productive clones under all light treatments, respectively (Figure 2C). The low productive clones generally showed CHL_A/B_ < 3.5, which is indicative for shade conditions. However, the responses of the clones with intermediate field performance were heterogeneous, particularly under RB and RGB treatments, which had low Y and O intensities (Table 3). Under RB treatment, which lacked green light, clones No. 28, 84, 86 showed increased CHL_A/B_, which was comparable to the top-performing clones (Figure 2C). In contrast, clones No. 90 and 97, which were the most productive among the intermediate ones, were not sensitive to light treatments, showing decreased CHL_A/B_, which was comparable to that of low productive clones.

## 3. Discussion

Illumination is known to have carry-over (lasting) effects on morphology and anatomy of leaves [53], which in the case of hybrids, are likely involved in heterotic interactions [8,9,10]. The effects of heterosis have been mainly studied on field performance [8], which result from complex synergic interactions of physiological and morphological traits [9,13], likely contributing to the observed clone-specific responses to light treatments (Table 2). The first two principal components of the studied traits of the experimental units (Figure 1B), however, indicated that only a specific combination of traits could be associated with superior field performance, as expected for heterotic genotypes [8,54]. Nevertheless, the performance of the statistical models (marginal R^2^ ≥ 0.63; Table 2), which is high for biological systems, implied explicit systematic effects of clone and treatment, confirming sufficiency of the experimental setup for comprehensive assessment of responses of different plant reproductive material to illumination [20,49].

Parental species of the studied hybrid aspen can regenerate vegetatively by root suckers [55], and these saplings are largely assimilating on G light, which has the best penetration through canopy [38,43]. Hence juvenile shoots of aspen can be shade tolerant as an adaptation for successful advanced growth and for colonization of potential canopy openings [56]. In contrast, R light is abundant under open conditions [57], in canopy openings, thus supporting the observed sensitivity of plantlets in vitro to these spectral regions of illumination (Table 2, Figure 2). However, due to the necessity to rapidly switch between such two such conditions, the signaling pathways are linked and interact [58]. The effects of illumination of specific spectral composition on the development of trees in vitro have received some attention [19,21], although LED illumination of wider spectral composition has been estimated with positive, yet genotype-specific effects on *Populus* spp. compared to monochromatic and FL light [19,20,59,60]. The observed responses of the plantlets to light of composed spectrum (Table 2, Figure 2) indicated complexity of signaling pathways [26,61], suggesting the biasness of responses to monochromatic light [36,37].

The complexity of the signaling pathways in response to the spectral composition of light could be related to shade tolerance and avoidance, which drives growth and has been linked to the amount of R and FR, B and UV-A light, and the B:G light ratio [25,40]. Although absorption of R rather than B light by chlorophyll is known to be more effective [62], the combination of both had up-regulatory effects on the efficiency of photosynthesis for hybrid aspen, as indicated by the individual effect of light treatment (particularly RB light) on CHL_A/B_ (Figure 2C). In contrast, CHL_A+B_ was increased under the FL (Figure 2B), which had the lowest amount of R and B:G, yet the highest amount of O light (Table 3), supporting the general compensatory mechanism by increasing the size of photosynthetic apparatus [62]. The responsiveness of the clones of hybrid aspen to light treatments differing O and Y lights (Table 3; Figure 2), which penetrate canopy well, suggested even more complex regulation of shade avoidance [43]. Furthermore, the clone-by-treatment interaction estimated for the studied traits (Table 2, Figure 2) indicated that the mechanisms of shade tolerance and avoidance were genotype-specific [63].

Boosted field performance and competitiveness has been linked to decreased environmental sensitivity due to allocation of assimilates to growth [8], while reducing defenses and reserves [64]. Accordingly, the morphology and physiology of the top-performing clones, as shown by TLA, as well as CHL_A+B_ were little affected by the experimental light treatments alone (Figure 2A,B). Such a strategy provides explicit advantages in the short-term, however, it increases environmental susceptibility in the long run [65], making the effects of heterosis unstable [9,14]. Moreover, superior field performance has been linked with efficiency of photosystems (e.g., CHL_A/B_) rather than their size (e.g., CHL_A+B_, TLA), thus facilitating structural growth and reproduction [15,66,67], while allocating minimal investments in photosynthetic apparatus (TLA). Accordingly, CHL_A/B_ as an indicator of adaptability to light conditions [30,62] and potential productivity [34], was increased in the top-performing clones (Figure 2C). Nevertheless, the CHL_A/B_ of the top-performing clones was sensitive to spectral composition of illumination (Figure 2C), indicating the ability to fine-tune their photosystems [44,47], which contributes to the field performance [66,67]. The top-performing clones increased CHL_A/B_ in response to illumination with wider spectral composition, particularly with Y and O light (FL and RGBYO, Figure 2C), thus indicating the ability to rapidly adjust to open conditions [21], which could affect further development ex vitro [59]. Such effects, though, can be transient due to genotype specific upregulation of chloroplast targeted genes [64,68].

The heterotic F1 hybrids can excel in assimilation due to increased TLA [8], which apparently was explicit for vegetative growth of some of the intermediately productive clones (Figure 2A, Table 2). Likewise, specific responses of other traits were estimated for hybrid aspen, particularly with intermediate field performance (Figure 2). Nevertheless, CHL_A/B_ was also high for some clones of intermediate productivity under RB light treatment (Figure 2), highlighting clonal differences [14,17]. Clones with intermediate and low field performance were more sensitive to light signals related to shade avoidance, particularly G and R light, as indicated by contrasting values of the selected variables (Figure 2), which suggests a diversification of the shade avoidance mechanisms. This implies that the latter might have the potential to be grown outside monocultures [15], where synergic interactions of traits of different genotypes can substantially contribute to productivity and sustainability [69,70]. Some of the less productive clones have also lost the sensitivity to shade avoidance signals [25,41], as indicated by low CHL_A/B_ irrespectively of light treatment, given that the illumination intensity was rather high.

It must, though, be admitted that the obtained results represent plantlet photomixotrophical growth, which is affected complexly by light, sugar, and auxin signals [71,72]. Hence the photomixotrophical propagation might have interfered with the effects of illumination, restricting the generalization of the responses to subsequent stages of propagation [73,74,75]. Furthermore, jar, which represents the experimental setup, had a notable effect on CHL_A+B_ and CHL_A/B_ (random variances, Table 2), which might be explained by intraspecific competition stratifying “canopies”, to which both variables are indicative [76]. Such effects were negligible (Table 2) for the plantlets grown in solitary test tubes, supporting the influence of competition [76] and suggesting application of single type of glassware.

## 4. Materials and Methods

### 4.1. Experimental Setup

Under controlled environment, morphological and physiological responses of in vitro cultures of clones of hybrid aspen differing by productivity were experimentally tested in four light treatments. In a climate chamber maintained at 25 °C and 30–40% relative humidity, four multi-layer shelve systems with a shelve size of 120×100 cm and height of 35 cm were equipped with luminaries. The luminaries were placed at 30 cm height above the shelves. The shelve systems were separated by non-transparent screens to avoid light contamination from others.

**Figure 3:**
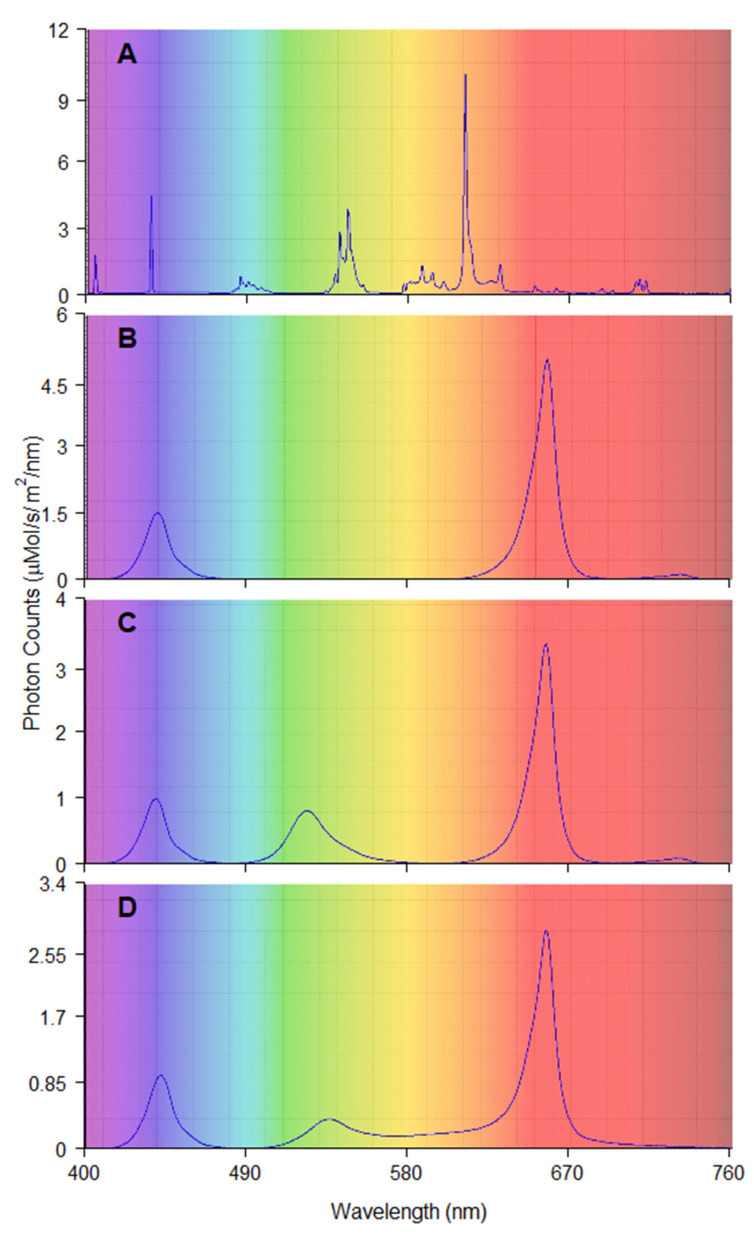
Spectral composition and photon count of each lighting treatment: (**A**) fluorescent tubes (FL); (**B**) Red and Blue (RB), max photon count at 655 and 440 nm; (**C**) Red, Green and Blue (RGB) max photon count at 655, 520, 440 nm; (**D**) Red, Green, Orange, Blue (RGBYO), max pho-ton count 655, 535, 625, 445 nm.

Three LED light treatments of (1) red, green, blue, yellow, and orange (RGBYO); (2) red, green, and blue (RGB); (3) and red and blue (RB) (Table 3) were tested to assess the synergic effects of different parts of spectrum on the performance of plantlets in vitro [20,27,30,58]. Fluorescent light (FL) from conventionally used luminescent tubes Philips Master TL-D 36 W warm white was used as the control treatment (Figure 3). The RB and RGB (LED) treatments were supplemented with far-red diodes, thus providing the spectral region of phytochrome absorbance [77,78]. This region in RGBYO treatment was provided by the yellow diodes, which emit light of wider spectrum. The studied LED light treatments had the red and blue ratio (R:B) of 3.2:1 and the red and far red ratio (R:FR) within the range of 28–36:1. The R:B and R:FR ratios for the FL treatment were 0.24:1 and 3:1, respectively, distinguishing it from others.

Irrespective of light treatment, the density of the photon flux of 110 ± 10 µmol m^−2^ s^−1^ for the wavelength ranging 400–750 nm with 16/8 h light/dark period imitating long day condition of growing period [79] was maintained. The homogeneity of illumination intensity was verified prior to the experiment, calibrating luminaries according to the light conditions measured at a 10 × 10 cm grid on the shelves. Measurements were done using the AvaSpec ULS2048 spectrometer (Avantes, Apeldoorn, The Netherlands). Adjustments were done when necessary.

### 4.2. Plant Material

Progenies of controlled crossing of local plus trees of common aspen (*Populus tremula*) from local populations from the central and eastern parts of Latvia and American aspen (*Populus tremuloides*) growing in a botanical garden in the central part of Latvia were studied [80]. The crossings were made during the 1960s on cut branches; the seeds were raised in local nurseries and two-year old bare-rooted seedlings were used for establishment of progeny trials [80]. The selection of trees with the best field performance for vegetative propagation was done at the age of 20 years; afterwards clonal plantations were established on former agricultural land [81]. The clonal plantations had randomized block or singletree plot design. In 2015–2017, meristem cultures from the clones growing in a trial in the central part of Latvia were collected from the lower branches at the age of 19–22 years for permanent maintenance in an in vitro clone archive.

To evaluate the relationships between sensitivity to illumination treatments and field performance of aspen hybrids, nine clones with differing productivity were selected. The selection was based on consolidated rankings by standing volume across trials in Latvia at the age of 18–35 years. Clones No. 5 and 24 were selected for representation of slow growing genotypes, as their field performance was lower compared to common aspen at similar age [82]. In contrast, clones No. 4 and 44, which performance greatly exceeded common aspen, were selected for the representation of the fast-growing genotypes. Clones No. 28, 84, 86, 90, and 97 had an intermediate performance, which slightly exceeded that of parental species, yet had superior stem quality (straightness and branchiness), were selected to complement the field performance gradient. Such selection was done also to assess the variability effects of heterosis on responses of genotypes to light treatments [82].

Prior to exposition to the experimental light treatments, plant material has been maintained and propagated in vitro for ca. five–six years in the plant physiology laboratory of LSFRI Silava as a long-term clonal collection. The plant material was cultivated on Murashige and Skoog medium (MS) [83], supplemented with MS micronutrients, MS vitamins, 0.1 mg L^−1^ idole-3-butyric acid (IBA), 20 g L^−1^ of sucrose, and 6 g L^−1^ agar (Sigma–Aldrich, St. Louis, MO, USA). The pH of the medium was maintained at 5.8. In the clone collection, all plantlets were growing under the same illumination provided by Philips Master TL-D 36 W florescent tubes, emitting photon flux density of 110 ± 10 µmol m^−2^ s^−1^. The material for the test was propagated according to routine maintenance protocol.

For each clone, eight 1.5 cm long apices were excised and transplanted into 300 mL jars filled with 30 mL of the same plant media, as used for preceding cultivation; jars were sealed with aluminum foil according to common practice due to convenience of preparation of media (sterilization) and optimal air exchange (Supplementary material, Figure S1A). For each light treatment and clone, 20 jars were prepared (720 jars in total). Additionally, for morphometrics, plantlets were cultivated solitarily in 40 mL test tubes filled with 5 mL of plant media and plugged with a cork allowing sufficient air exchange (Supplementary material, Figure S1B). For each clone and light treatments, 30 test tubes were prepared (1080 test tubes in total). In total, 6840 plantlets were subjected to light treatments. Jars and test tubes with plantlets were randomized in shelves/holders and subjected to the experimental light treatments for 30 days. The spacing between jars was 5 cm. Test tubes were randomly placed into test tube holders. The experiment was conducted in May 2021.

### 4.3. Measurements

Morphological and physiological variables were measured for the assessment of the effects of illumination treatments on plantlets of hybrid aspen. For 26–30 solitarily grown plantlets per treatment/clone (the numbers deviate from the prepared due to mortality), the length of the main and lateral shoots (excluding initial length of the explant) was measured, and the number of internodes and lateral shoots were recorded. The largest and best developed axis was considered as the main shoot. Swelled lateral buds shorter than 0.5 cm were not considered as a lateral shoot. Total length was considered as the sum of lengths of all shoots. Additionally, the length of the third internode, which is considered to show the strongest effect of the treatment [84], was measured.

For measurements of leaf area, all the leaves (without petioles) formed after explanting (leaves of the lowest node were excluded) were levelled and fixed on a transparent glass, and high-resolution grayscale images (1275 × 1752 px) at 24-bit color depth were acquired using Canon LC4800P scanner (Canon Inc., Tokyo, Japan). The images were processed using WinFolia Pro 2019 (Regent instruments Inc., Québec City, Québec, Canada). Leaf deformations (cracks, gaps, overlaps etc.) were corrected using editing options provided by the software and further analysis was conducted on edited images. Area of individual leaves, mean, and total leaf area, was determined by selecting leaves of each separate plant as separate measurement regions. Leaf recognition was based on color differences in selected regions and for majority of samples default set point of program was used. The number of leaves and their mean and total area were counted/measured for each plantlet.

Concentration of pigments, chlorophyll a and b, fluorescence intensity and enzymatic activity was measured for the assessment of physiological responses of in vitro plantlets of hybrid aspen to light treatments. To determine concentrations of photosynthetic pigments (chlorophyll a and b, total chlorophyll, and total carotenoids), five jars were randomly taken for each light treatment/clone. All the leaves formed during the light treatment were taken and homogenized using Retshc MM400 (Retsch GmbH, Haan, Germany) ball mill; 125 mg of the homogenate were taken. Pigments were extracted in 5 mL of pure acetone (99.7%) supplemented with 15 mg CaCO_3_ [85]. The extract was centrifuged for 10 min at 7000 RPM at +4 °C, supernatant was stored in fridge. Optical density at 662.5; 644.5 and 470 nm wavelength was measured to assess pigment concentration using Lambda 25 UV/VIS (PerkinElmer Inc., Akron, Ohio, USA) spectrophotometer and UV Winlab v2.85.04 (PerkinElmer Inc., USA) software. For this 0.75 mL of supernatant were diluted with 2.25 mL of acetone in a quartz cuvette.

To measure relative chlorophyll concentration, another five random jars per treatment/clone and five random plantlets per jar were selected (25 plantlets per unit). For each plant, measurements were made on the third youngest leaf in three to five points using Minolta SPAD (Konica Minolta Sensing Inc., Tokyo, Japan) chlorophyll meter and averaged for leaf. The efficiency of photosystem II, fluorescence of chlorophyll a was measured using HandyPea+ (Hansatech Instruments Ltd., UK) fluorometer on the third youngest leaf following standard procedure [86]. Three plants from five jars were randomly selected (15 plantlets) per treatment/clone. A red light pulse of 1 s and 1500 umol m^2^ s^−1^ intensity was used; maximum quantum yield efficiency (F_v_/F_m_) and performance or vitality index (PI_ABS_) were assessed by PEA PLUS v.1.13 (Hansatech Instruments Ltd., Norfolk, UK) software.

The activities of peroxidase (POX) and catalase (CAT) were measured as the measures of biochemical processes according to Andersone and Ievinsh [87]. Five jars per clone/treatment were taken and 175 mg of freshly formed leaves (sampling from the apex) were collected and homogenized. The homogenate was centrifuged for 20 min at 13,000 RPM and 4 °C; total protein concentration was determined using Pierce Detergent Compatible Bradford Assay Kit (Thermo Fisher Scientific, New York, USA) according to Bradford [88]. The activity of POX and CAT was determined using Lambda 25 UV/VIS spectrophotometer (PerkinElmer Inc., Boston, MA, USA) and UV Kinlab v2.85.00 software (PerkinElmer Inc., USA). For POX, supernatant (20 µL) was filled in a plastic cuvette with 2 mL of sodium buffer (pH 7.0) with guayacol, then 0.5 mL of 30 mM H_2_O_2_ was added. The reaction solution without H_2_O_2_ was used as a reference. Absorption changes at 470 nm wavelength were registered each 2 s for 2 min. For CAT, 2.25 mL of the buffer solution at pH 7.8 were poured in a quartz cuvette and mixed with 0.750 mL 30 mM H_2_O_2_, then 50 μL of supernatant was added. Absorption changes were registered each 2 s for 1.5 min. The buffer solution without supernatant was used as a reference. The activities of POX and CAT were expressed as absorption changes per 1 g protein.

### 4.4. Data Analysis

The concentration of chlorophyll a, b, and total, as well as carotenoids were calculated according to Lichtenthaler [89]:

CHL_A_ = 11.24 × A662.5 − 2.04 × A644.5, (1)

CHL_B_ = 20.13 × A644.5 − 4.19 × A662.5, (2)

CHL_A+B_ = 7.05 × A662.5 + 18.09 × A644.5, (3)

CAR = (1000 × A470 − 1.90 × CHL_A_ − 63.14 × CHL_B_)/214, (4)

where A—absorption at corresponding wavelength; CHL_A_—chlorophyll a concentration; CHL_B_—chlorophyll b concentration; CHL_A+B_—total chlorophyll concentration; CAR—total carotenoid concentration. Pigment concentrations were expressed as µg mL^−1^ of diluted extract. Additionally, the ratio of concentration of chlorophyll a and b was calculated. To assess the main patterns of variation in the dataset, which consisted of several variables, Principal Component Analysis (PCA) was performed as the data reduction technique. As the measurements were made at different levels (leaves and plants) and scales, the PCA was based on scaled (z-score) mean values for clones/treatments (experimental units). The analysis was based on the correlation matrix.

The effect of light treatment and clone on the response variables representing the principal components of the datasets were assessed using a linear or generalized linear model according to the type of data analyzed. Generalization according to Poisson distribution of the residuals was used for the discreet measurements (e.g., count data). Models were based on the non-aggregated measurements of variables. The models in general form were as follows:

Y = μ + LED + C+ LED×C + RE + ε,(5)

where LED—the effects of light treatment (four levels); C—the effect of clone (nine levels); LED×C—the effect of clone by treatment interaction, and RE—the random effects of experimental design (if appropriate). The simple or nested multilevel structure of random effects was used according to levels of measurements (i.e., jar, plant, and leaf for the physiological variables) or random effects were not used for solitary jar/test tube measurements. The mixed models were fitted using the restricted maximum likelihood approach. Model assumptions and residuals were checked by the diagnostic plots. The Wald’s type II χ^2^ test was used to assess the significance of the fixed effects. For the levels of significant fixed effects, estimated marginal means were compared pairwise by the Tukey’s Honest Significant Difference test. Data analysis was conducted in the software R, v. 4.2.1 [90] using packages lme4 [91] and emmeans [92].

## 5. Conclusions

Clone specific sensitivity of hybrid aspen to illumination at the early stages of development in vitro emphasizes the necessity for specific adjustments in illumination for clones and/or clone groups to increase the efficiency of propagation, particularly considering later (ex vitro) stages. The complex responses to multispectral light (Table 2) indicated the necessity for more elaborate adjustments of in vitro cultivation protocols, targeting specific genotypes to maximize the overall efficiency of the entire process [58]. In addition, light of wider spectral composition has been shown to be more efficient for propagation of plant material that is more vigorous and agile [93]. The extended spectral composition of illumination generally increased TLA, which from the practical point of view suggests that plantlets might be able to cope with more mechanical damage to leaves during the everyday maintenance of the in vitro cultures. Under the warming climate in temperate and, particularly, in the hemiboreal zone the extended vegetation season of hybrid aspen is linked to frost damage [94], clones with lower field performance, yet with more elaborate regulation of physiological and morphological processes, apparently, might become more viable alternatives for commercial application. Considering that multi-clone plantations of hybrid aspen are recommended to reduce environmental risks—an increasing demand for different genotypes [84], plasticity of luminaries in terms of adjustment of spectral composition appears to be crucial to maximize the efficiency of the propagation.

## Figures and Tables

**Table 1:** Descriptive statistics of the measured morphological and physiological variables of in vitro plantlets of hybrids of aspen differing by productivity under the studied light treatments. The statistics were calculated based on the mean value of the experimental units (clones and light treatments).

Variable	Abbreviation	Range	Mean	Median	St. Dev.	Coef. of Variation
Number of internodes	NI	4.25–10.48	6.37	5.90	1.80	0.28
Length of third internode	LTI	1.71–4.8	3.41	3.46	0.91	0.27
Main shoot length	MSL	1.34–3.41	2.34	2.24	0.67	0.28
Total shoot length	TSL	2.91–5.24	4.07	4.00	0.73	0.18
Mean leaf area	MLA	0.28–0.84	0.61	0.64	0.12	0.20
Total leaf area	TLA	2.93–11.32	6.55	5.78	2.64	0.40
Concentration of carotenoids	CAR	2.78–5.54	3.89	3.80	0.64	0.19
Concentration of chlorophyll a	CHL_A_	8.59–20.11	12.50	12.48	2.53	0.20
Concentration of chlorophyll b	CHL_B_	1.81–7.07	3.85	3.94	1.15	0.30
Total chlorophyll concentration	CHL_A+B_	10.5–27.2	16.66	16.55	3.96	0.24
Chlorophyll a and b ratio	CHL_A/B_	2.72–4.6	3.37	3.17	0.55	0.16
Maximum quantum yield efficiency	F_v_/F_m_	0.56–0.81	0.74	0.77	0.07	0.10
Performance index	PI	0.29–3.56	1.47	1.24	0.99	0.67
Catalase activity	CAT	1.08–4.06	1.95	1.87	0.67	0.34
Peroxidase activity	POX	0.34–5.12	1.40	1.19	1.07	0.76

**Table 2:** The fixed effects (Wald’s χ^2^ and *p*-value) of clones, studied light treatments of different spectral composition and their interaction on morphological and physiological variables of plantlets of hybrid aspen in vitro, the variances of random effects of experimental setup (according variable), and overall performance (R^2^ values) of the mixed effects model. Significance code: ***—*p*-value < 0.001. For random effects, interclass correlation (ICC) is show in brackets.

Concentration of Chlorophyll a and b	
***Fixed Effects, χ^2^***	
Light treatment	26.26 ***
Clone	229.47 ***
Light by clone interaction	83.33 ***
***Random effects, variance, and ICC***	
Jar	7.50 (0.79)
Residual	1.97
***Model fit***	
R^2^ (marginal)	0.63
R^2^ (conditional)	0.92
**Chlorophyll ratio (a/b)**	
***Fixed effects, χ^2^***	
Light treatment	22.75 ***
Clone	766.65 ***
Light by clone interaction	107.43 ***
***Random effects, variance***	
Jar	0.050 (0.56)
Residual	0.039
***Model fit***	
R^2^ (marginal)	0.79
R^2^ (conditional)	0.91
**Total area of leaves**	
***Fixed effects, χ^2^***	
Light treatment	104.12 ***
Clone	2014.79 ***
Light by clone interaction	368.41 ***
***Random effects, variance***	
Test tube holder	0.15 (0.05)
Residual	2.67
***Model fit***	
R^2^ (marginal)	0.69
R^2^ (conditional)	0.72

**Table 3:** Spectral composition % of total photon flux (from 400 to 750 nm) for light treatments used in this experiment.

	Red and Blue (RB)	Red and Green and Blue (RGB)	Red and Green and Blue and Yellow and Orange (RGBYO)	Fluorescent Tubes (FL)
Blue 400–500 nm	23	18	17	17
Green 500–570 nm	0	22	17	25
Yellow 570–590 nm	0	0	3	7
Orange 590–625 nm	2	1	5	36
Red 625–700 nm	73	57	56	11
Far-red 700–750 nm	2	2	2	4
Red:Blue (R:B)	3.17	3.17	3.29	0.65
Red:Far-red (R:FR)	36.5	28.5	28	2.75
Blue:Green (B:G)	n/a	0.82	1.00	0.68

## Data Availability

The datasets generated during and/or analyzed during the current study are available from the corresponding author on reasonable request.

## Supplementary Materials

The following supporting information can be downloaded at: https://www.mdpi.com/article/10.3390/plants11202692/s1, Figure S1. Example of cultivation glass jars (A) and test tubes (B) used for propagation of hybrid aspenplantlets. Aluminium caps are used for convenience of sterilization as well due to antiseptic properties.
