# Supplementary material for: Morphological and Physiological Responses of Hybrid Aspen (Populus tremuloides Michx. × Populus tremula L.) Clones to Light In Vitro
Source: Plants (Basel). 2022 Oct 12;11(20):2692. doi: 10.3390/plants11202692 (PMC9607416; doi:10.3390/plants11202692)
Supplement: Supplementary file 1 [file plants-11-02692-s001.zip › plants-1955334-supplementary.pdf]

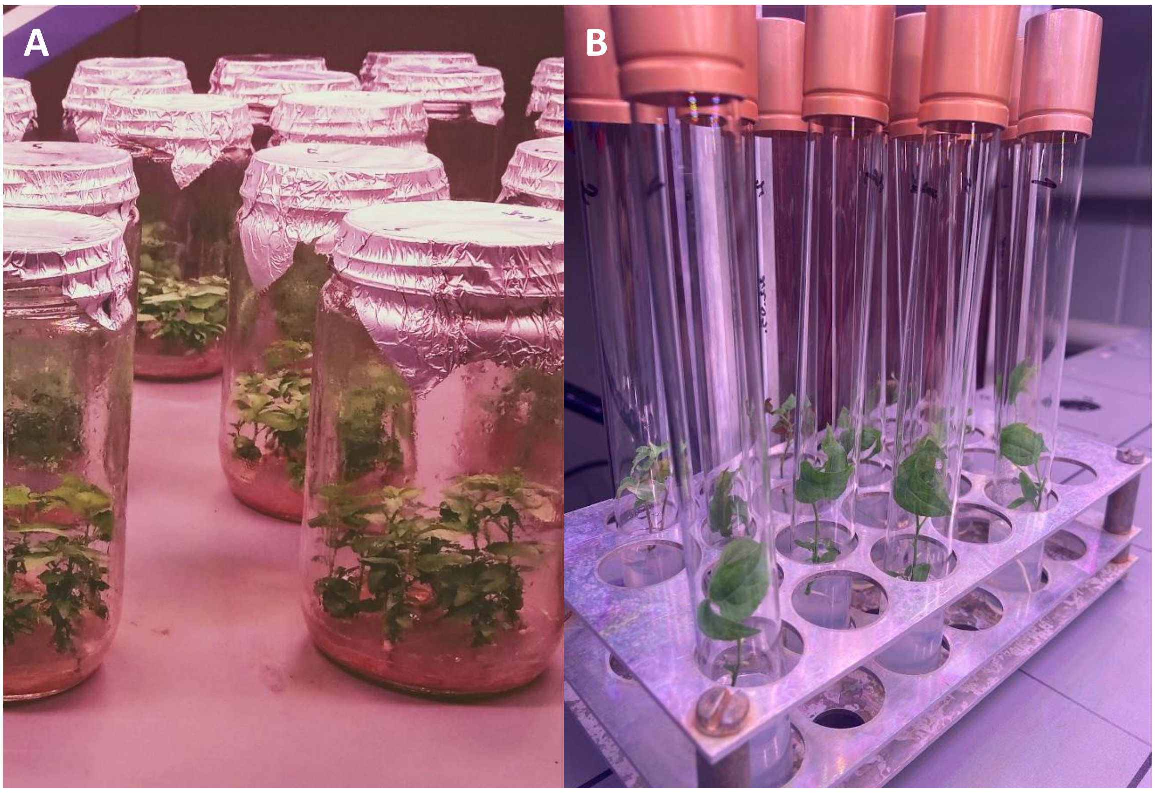

Figure S1. Example of cultivation glass jars (A) and test tubes (B) used for propagation of hybrid aspen plantlets. Aluminium caps are used for convenience of sterilization as well due to antiseptic properties.
